# Fluid-Phase Endocytosis and Lysosomal Degradation of Bovine Lactoferrin in Lung Cells

**DOI:** 10.3390/pharmaceutics14040855

**Published:** 2022-04-13

**Authors:** Edward John Sayers, Iwan Palmer, Lucy Hope, Paul Hope, Peter Watson, Arwyn Tomos Jones

**Affiliations:** 1 School of Pharmacy and Pharmaceutical Sciences, Cardiff University, King Edward VII Avenue, Redwood Building, Cardiff CF10 3NB, Wales, UK; sayersej@cardiff.ac.uk (E.J.S.); palmerg2@cardiff.ac.uk (I.P.); 2 Cardiff School of Biosciences, Cardiff University, Museum Avenue, The Sir Martin Evans Building, Cardiff CF10 3AX, Wales, UK; watsonpd@cardiff.ac.uk; 3 Virustatic, M-SParc, Gaerwen, Isle of Anglesey LL60 6AG, Wales, UK; lucyhope@virustatic.com (L.H.); paulhope@virustatic.com (P.H.)

**Keywords:** lactoferrin, endocytosis, intracellular trafficking, lysosomal degradation

## Abstract

The iron-binding protein lactoferrin and the cell-penetrating peptides derived from its sequence utilise endocytosis to enter different cell types. The full-length protein has been extensively investigated as a potential therapeutic against a range of pathogenic bacteria, fungi, and viruses, including SARS-CoV-2. As a respiratory antiviral agent, several activity mechanisms have been demonstrated for lactoferrin, at the extracellular and plasma membrane levels, but as a protein that enters cells it may also have intracellular antiviral activity. Characterisation of lactoferrin’s binding, endocytic traffic to lysosomes, or recycling endosomes for exocytosis is lacking, especially in lung cell models. Here, we use confocal microscopy, flow cytometry, and degradation assays to evaluate binding, internalisation, endocytic trafficking, and the intracellular fate of bovine lactoferrin in human lung A549 cells. In comparative studies with endocytic probes transferrin and dextran, we show that lactoferrin binds to negative charges on the cell surface and actively enters cells via fluid-phase endocytosis, in a receptor-independent manner. Once inside the cell, we show that it is trafficked to lysosomes where it undergoes degradation within two hours. These findings provide opportunities for investigating both lactoferrin and derived cell-penetrating peptides activities of targeting intracellular pathogens.

## 1. Introduction

Lactoferrin (LF) is a highly conserved, cationic glycoprotein of the transferrin family, which is synthesised by exocrine glands and secreted by neutrophils in infection and inflammation sites [1]. Several studies have shown that LF can internalise into cells, and a number of suggested receptors have been implicated in this process [2], taking the protein via, for example, clathrin-coated vesicles to different organelles [3]. The N-terminus of human LF (hLF) has a 22 amino acid sequence that was identified as a cell-penetrating peptide (CPP) that, when examined as a standalone peptide, utilises endocytosis to gain cell entry [4]. This sequence (38-KCFQWQRNMRKVRGPPVSCIKR-59) sits as a member of a huge list of naturally derived and artificial CPPs that have the ability to overcome the plasma membrane of cells to gain entry, either via direct translocation, or by hijacking one or more endocytic pathways [5]. Classical examples include the Tat peptide and penetratin, which are derived from the HIV-Tat protein and *Drosophila* antennapedia, respectively; synthetic variants such as octaarginine have also been described as CPPs.

Despite being highly conserved, there are significant differences in the amino acid sequences of LF from different species [6] which could impact cell binding and uptake; including in the aforementioned N-terminus. hLF contains the N-terminal sequence GRRRR, which, as a pentapeptide, was found to be crucial for heparin binding [7] and can be internalised rapidly and localised to the nucleus in a number of cell lines [8]. A pentapeptide derived from this region of bovine LF (bLF) (40-RRWQW-44) has been also shown to act as a CPP and is able to internalise plasmid DNA into A549 (human alveolar basal epithelial cells) [9]. A 25-amino-acid peptide from bLF (37-FKCRRWQWRMKKLGAPSITCVRRAF-61), equivalent to the hLF peptide described above (residues 38–59), has also been shown to rapidly internalise in a range of human breast cancer cells, remaining in the cytoplasm and not entering the nucleus [10].

The properties of LF and its peptide derivatives have been utilised as a delivery mechanism, either using the protein/peptide directly [9,11] or using the protein as a targeting/cell binding entity (reviewed here [12]). For instance, hLF along with chitosan have been conjugated to polyethyleneimine polyplexes to deliver miRNA into the brain [13], while prostate cancer cells have been targeted using LF as the targeting entity of gold nanocages carrying plasmid DNA [14].

Lactoferrin is currently of significant interest as it plays an important role in host defence against viral attack [15,16]. It can directly bind to viral proteins [17,18,19], and bind to heparan sulphate proteoglycans (HSPGs) on the host cell surface [20,21], with both mechanisms inhibiting viral attachment to, and entry into, target cells. In the context of CPPs, many utilise HSPGs to gain cell entry to endosomes, and bLF has been shown to disrupt the intracellular trafficking of viruses [22], which may well occur at the level of the endosome.

In vitro studies have shown that bLF inhibits early-phase viral infection by several human coronaviruses [20,23], including SARS-CoV-2, in a range of cell types [24,25,26]. Given the ongoing COVID-19 health crisis caused by the SARS-CoV-2 virus, there has been much interest in the role LF plays as a broad-spectrum antiviral in the management of COVID-19 patients, and also as a prophylactic treatment [27]. However, more research into the intracellular trafficking of bLF is required for this potential to be realised.

Very few recent studies have investigated in detail the endocytosis of intact bLF, despite being more easily available as a natural glycosylated protein than hLF, and thus having important therapeutic potential. In this study, we performed detailed endocytosis, trafficking, and intracellular stability analyses of a fluorescent conjugate of bLF. Our studies show that the protein binds to the surface of lung A549 cells but is displaced by washing with high concentrations of negatively charged heparin. These cells do not express the lactoferrin receptor, and bLF is subsequently endocytosed through the fluid-phase where it is trafficked to the lysosome and degraded within two hours. These studies should pave the route to further analysis of the corresponding CPP sequence derived from this protein, allowing better understanding of its mechanism of internalisation and potential as a cell-penetrating moiety that may also have other therapeutic applications, beyond the infectious disease space.

## 2. Materials and Methods

### 2.1. Materials

Bovine lactoferrin (bLF, Ingredia, Arras, France) was dissolved in distilled water to either 10 mg/mL or 20 mg/mL, and was passed through a 0.22 µm syringe filter for sterilisation. Reconstituted bLF was stored at −20 °C and used within 2 weeks.

Triton X-100, Tween-20, heparin, glycine, sodium dodecyl sulfate (SDS), and sodium bicarbonate were purchased from Sigma Aldrich, Gillingham, UK. Staurosporine, Alexa647 succinimidyl ester, transferrin-alexa488 (Tf488), Dextran-alexa488/-alexa647 (Dex488 or Dex647), and BSA (fraction V) were obtained from Fisher Scientific, Loughborough, UK.

### 2.2. Cell Culture

Human epithelial lung cells, A549 (CLL-185), were routinely cultured in DMEM (Fisher Scientific, Loughborough, UK) containing 10% foetal bovine serum (Thermo Fisher, Paisley, UK) under tissue culture conditions (37 °C, 5% CO_2_ in a humidified incubator). Cells were passaged twice weekly using 0.25% Trypsin/EDTA (Fisher Scientific, Loughborough, UK) for a maximum of 20 passages from defrosting and kept below 80% confluency during routine cell culture. For all experiments, cells were seeded at 15.8 × 10^3^ cells.cm^−2^ in complete medium (DMEM + 10% FBS) under tissue culture conditions, with the final timepoint of 48 h after seeding.

### 2.3. Labelling bLF with Alexa647

bLF was labelled using the method described previously [28]. Briefly, bLF was reacted with Alexa647 succinimidyl ester at a molar ratio of 1 to 4 for 4 h in 0.1 M sodium bicarbonate buffer pH 8.0. Unreacted dye was removed, and the protein transferred into PBS by successively passing through two Zeba spin desalting columns (Sigma Aldrich, Gillingham, UK) preloaded with PBS according to the manufacturer’s instructions. The protein concentration and degree of labelling was calculated by UV-Vis absorbance (Agilent, Stockport, UK), measuring Alexa647 at 650 nm and the Alexa647-compensated protein peak at 280 nm, giving a final concentration of 23.07 µM bLF647, with an average of 1.93 moles fluorophores per mole protein. The bLF647 was filter-sterilised before use.

### 2.4. Viability Assays

A549 cells were seeded in 75 µL complete medium per well in a black, flat bottomed, 96-well tissue-culture-treated plate (Fisher Scientific, Loughborough, UK) and incubated under tissue culture conditions for 24 h. Cells were then treated by mixing in an additional 25 µL of drug/diluent control in complete medium. bLF was added to the cells at a maximal final concentration of 4 mg/mL and as a positive toxic control, staurosporine was alternatively added at a maximum final concentration of 50 µg/mL, and both were serially diluted to 1:1 in complete medium; 0.2% Triton X-100 was used as an additional positive control for cell death. Cells were incubated for 20 h under tissue culture conditions before 20 µL CellTitre Blue (Promega, Southampton, UK) was added and returned to tissue culture conditions for 4 h. Fluorescence intensity was measured using a plate reader (Tecan, Theale, UK). A one-way ANOVA was performed to determine significant changes in viability.

### 2.5. Uptake Experiments

Following a 30 min starvation in serum-free medium (SFM), cells were incubated in 24-well plates with either 250 nM bLF647, 20 nM Tf488, or 50 µg/mL Dex647 in SFM for between 10 and 360 min and analysed by flow cytometry. Alternatively, cells in 24-well plates were incubated for 15 min with between 5 and 250 nM bLF647 or Tf488, or between 5 and 250 µg/mL Dex647 in SFM following a 30 min starvation, and were analysed by flow cytometry.

### 2.6. Cell Binding Experiments

Cells grown in 24-well plates or imaging dishes were chilled on ice for 10 min before washing and incubating with ice-cold 250 nM bLF647 in the presence of either 25 µM unlabelled bLF, 25 µM unlabelled Tf, 1 or 5 mg/mL Heparin for 1 h in SFM before being washed in SFM and analysed by flow cytometry. In addition, cells incubated with 250 nM bLF647 for 1 h were subsequently washed with 5 min washes of either 1 or 5 mg/mL heparin before washing in SFM and being analysed by flow cytometry or microscopy.

### 2.7. Endocytic Trafficking and Recycling Experiments

Cells were seeded into either 6-well plates (Western blotting), 24-well plates (flow cytometry), or imaging dishes (MatTek, Ashland, MA, USA, for confocal microscopy). The morning before the experiment, cells in imaging dishes were incubated with 100 µg/mL Dex488 or Dex647 for 3 h in complete medium under tissue culture conditions before being washed in complete medium and returned to tissue culture conditions to label late endosomes/lysosomes [28]. To perform the uptake/recycling assays, they were incubated with 500 nM bLF647 (flow cytometry and microscopy) or 1 µM unlabelled bLF (Western blotting) in SFM for 1 h before being washed in complete medium and incubated for a chase period of 0–24 h. Samples were analysed as described below.

### 2.8. Flow Cytometry

After treatment, cells were washed three times in PBS and lifted using Accutase (Thermo Scientific, Paisley, UK) for cell binding experiments, or 0.5% trypsin for other experiments. Cell detachment media was removed by centrifugation at 400× *g* for 2 min with the cells resuspended in ice-cold PBS (cell binding experiments), or ice-cold PBS-containing 2% FBS. Cells were subsequently analysed with a FACSVerse (BD Bioscience, Wokingham, UK) using the 488 or 633 nm laser lines for excitation. A single-cell population was obtained through double gating (FSC v SSC, then FSC-W v FSC-H), with the median readout obtained for population fluorescence intensity. A single experiment represents the mean of the median intensity value from two replicates (samples were run in duplicate). The final values represent the mean of at least three independent experiments.

### 2.9. Confocal Microscopy

After treatment, cells were washed three times in prewarmed/prechilled phenol-red-free DMEM (imaging medium) and imaged immediately on a Leica SP5 laser scanning confocal microscope. Images were obtained using a 100× 1.4 NA objective, raster size of 1024 × 1024, producing a pixel size of 137 nm. Where both Alexa488 (excited at 488 nm using Argon laser) and Alexa647 (excited at 633 nm using HeNe laser) were imaged, imaging was set to sequentially capture images between lines.

### 2.10. Quantification of Microscopy Images

Images were quantified using a custom macro in FIJI [29]. Data was also obtained for the total integrated density of the whole the bLF647, Tf488, or Dex647 channel. A minimum of 10 single sections were obtained per independent experiment with the images obtained 2 µm above the glass surface.

### 2.11. Western Blotting

Following the uptake experiments, cells were lysed using NP40 lysis buffer (Invitrogen, Paisley, UK)-containing protease inhibitors (cOmplete Mini, Roche, St Albans, UK), centrifuged at 11,000× *g*, and supernatant proteins were quantified in a BCA assay. Samples (30 µg) were mixed with loading buffer and loaded onto an anyKD SDS-PAGE gel (BD Biosciences, Wokingham, UK), and transferred onto PDVF using TransBlot Turbo (BD Biosciences, Wokingham, UK). Bands were detected using an anti-lactoferrin polyclonal, pre-conjugated to HRP (1:30,000, A10-126P, Cambridge Bioscience, Cambridge, UK) in 5% BSA/0.075% Tween 20/PBS for 1 h after blocking in 5% BSA/0.075% Tween 20/PBS for 1 h. The membrane was washed 3 × 20 min in 0.075% Tween 20/PBS and detected using chemiluminescence on a ChemiDoc (BD Biosciences, Wokingham, UK). The membrane was stripped using 2 × 10 min stripping buffer (15 g/L glycine, 1 g/L SDS, 0.01% (*v*/*v*) Tween 20, pH 2.2) followed by 2 × 10 min washes in PBS and 2 × 10 min washes in PBST (0.025% Tween 20/PBS). After reimaging on the ChemiDoc to check for removal, membranes were re-blocked in 5% BSA/PBST and incubated with Tubulin-HRP (1:50,000, ab21058, Abcam, Cambridge, UK) in 5% BSA/PBST. The membrane was washed 3 × 5 min in PBST, and the bands were detected using the ChemiDoc.

## 3. Results

**Figure 1:**
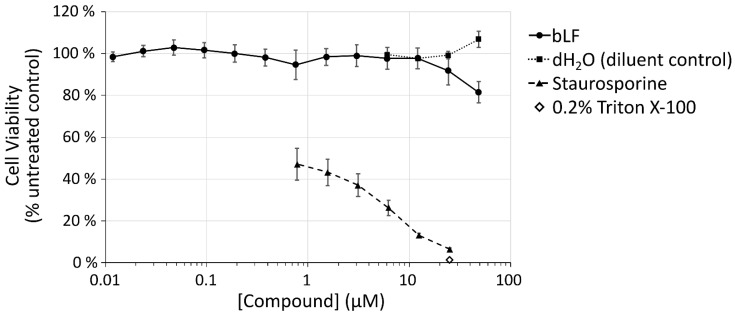
Viability analysis of bLF incubated with A549 over 24 h. A549 cells were incubated with bLF, diluent control (dH_2_O), staurosporine, or 0.2% Triton X-100 for 24 h in serum-containing medium before analysis using CellTitre Blue metabolic assay. Error bars represent SEM.

In A549 cells, a model cell line for the lower respiratory system, bLF is nontoxic up to 20 µM (Figure 1). Above this, there is a nonsignificant reduction in viability up to 50 µM (*p* = 0.23). This is in contrast to the control compound staurosporine, that showed a significant drop in viability when incubated with these cells (*p* < 0.0001).

**Figure 2:**
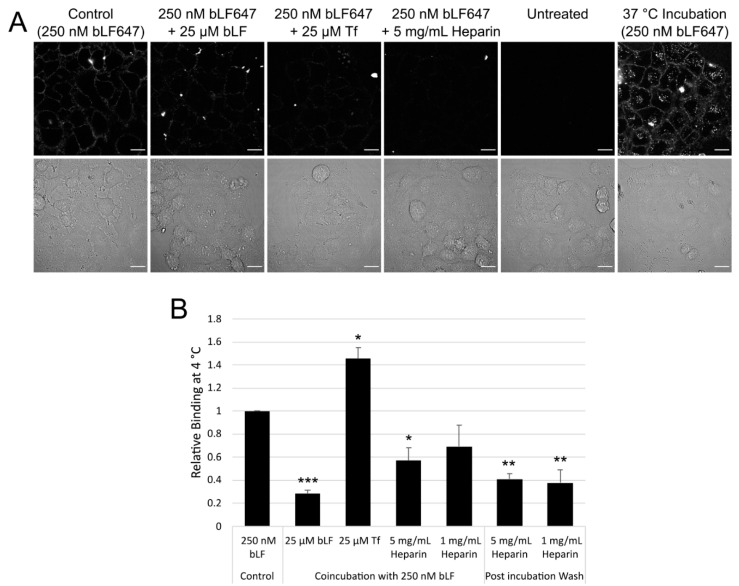
bLF cell binding and competition. Cells were incubated with 250 nM bLF647 in the presence of potential competitors for 1 h at 4 °C before being analysed by confocal microscopy (**A**) or flow cytometry (**B**). Scale bar = 50 µm, error bars represent SEM. Flow cytometry represents the mean of three independent experiments performed in duplicate; fluorescence intensity is measured using the median and normalised to the control. Statistical analysis was performed using a one-way ANOVA (F (6, 14) = 17.13, *p* < 0.0001) with a Dunnett post hoc analysis of control versus samples * *p* < 0.05, ** *p* < 0.01, *** *p* < 0.001.

Lactoferrin has been shown to interact with many different receptors [2], of which, the lactoferrin receptor (LfR) intelectin-1 is expressed predominately in the intestine, and is not shown to be expressed in A549 cells via RNAseq analysis [30] (Table S1 in Supplementary Materials). Other receptors implicated in endocytosis of this protein are, however, expressed in this cell line; therefore, we looked at the surface binding of bLF647 in the presence of various competitors. bLF647 was incubated with cells at 4 and 37 °C for 1 h and visualised by confocal microscopy or quantified by flow cytometry (Figure 2). At 4 °C, bLF647 showed moderate binding to the cell surface with no internalisation, whereas at 37 °C, punctate structures could be seen inside the cell, indicative of endocytosis. Coincubation with 100× excess of unlabelled bLF647 showed a significant decrease in the binding to the cell, while a 100× molar excess of unlabelled Tf showed an unexpected increase in plasma membrane bLf647 binding when analysed and quantified by flow cytometry. In comparison, and as expected, a 100× excess of unlabelled Tf completely inhibited Tf488 binding, while a 100× excess unlabelled bLF had no effect on Tf488 binding (Supplementary Figure S1).

Heparin sulphate proteoglycans have been implicated in the binding of LF and CPPs [2,31]. Coincubation with an excess of heparin or washing the cells after a 1 h incubation of bLF647 showed a significant reduction in binding, indicating roles for HSPG or surface charge in bLF binding.

**Figure 3:**
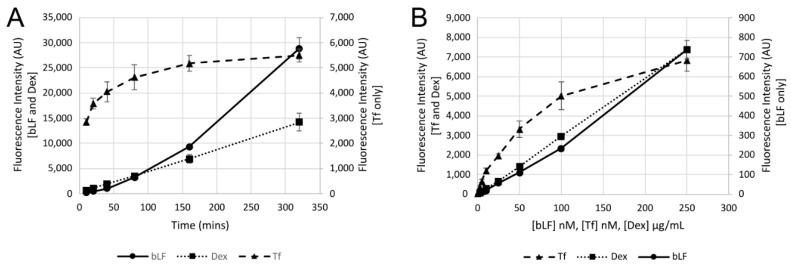
Kinetic analysis of the uptake of bLF647 in A549 cells. Uptake in A549 cells of bLF647, Dex647, and Tf488 analysed against time (**A**) or concentration (**B**). Cells were incubated with 250 nM bLF647, 50 µg/mL Dex647, or 20 nM Tf488 for between 10 min and 6 h; or cells were incubated with differing concentrations of bLF647, Dex647, and Tf488 for 15 min, and analysed by flow cytometry. Data represents the mean of three independent experiments with fluorescence intensity calculated as the mean from a duplicate of median cell intensity values. Error bars represent SEM.

The internalisation properties of bLF647 were compared with transferrin, a model for receptor-mediated endocytosis, and dextran, representing a model for fluid-phase endocytosis. Cells were incubated with bLF647 for different time periods and compared to Tf488 and Dex647 (Figure 3A). Transferrin was rapidly endocytosed into the cells before reaching a steady state after 160 min where uptake and recycling balance each other. Since fluid-phase endocytosis is an ongoing process, and not receptor-dependent, Dex647 endocytosis is a continuous process. Like dextran, bLF647 cell-associated fluorescence increased over the entire time period; however, the kinetic profile was very different. Cell uptake can also be compared as a function of concentration and assays were also conducted with increasing concentrations of the three analysed probes. Here, as in Figure 3B, fluid-phase endocytosis (Dex647) was linear with respect to concentration, while receptor-mediated uptake (Tf488) can be equated to receptor–ligand binding kinetics.

**Figure 4:**
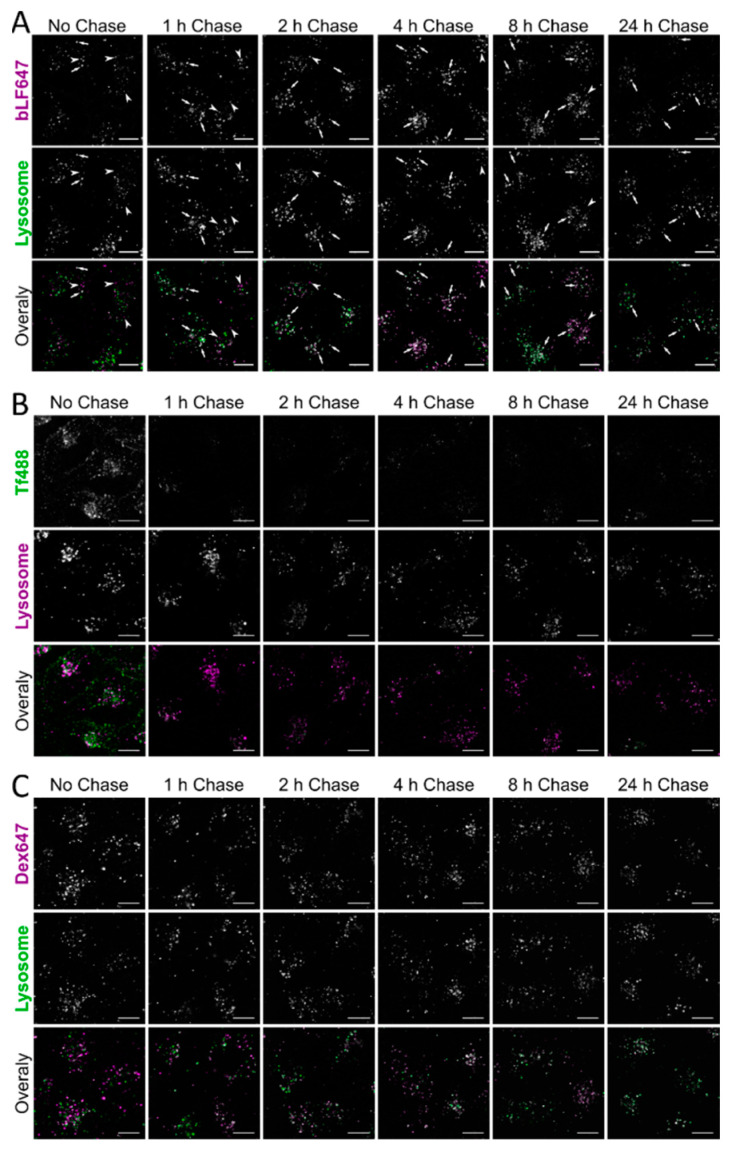
Trafficking of bLF647, Tf488, and Dex647 to lysosomes by confocal microscopy. Cells were incubated with 500 nM bLF647 (**A**), 20 nM Tf488 (**B**), or 100 µg/mL Dex647 (**C**) for 1 h in SFM, washed in serum-containing medium, and incubated for the chase period before being washed and imaged by confocal microscopy. Lysosomes (green, middle row) were prelabelled using a pulse–chase protocol. Arrows represent endocytic structures with colocalised bLF and endolysosomal structures (white), arrow heads indicate bLF647 endocytic structures not colocalised with the lysosome. Scale bar = 10 µm, representative images from three independent experiments quantified in Figure 5. See Supplementary Figures S2–S4 for uncropped images.

To determine the trafficking characteristics once bLF is endocytosed within the cell, the protein was pulsed for 1 h, chased, and imaged at intervals up to 24 h in cells with labelled lysosomes (Figure 4A). After 1 h, there was little evidence that bLF had reached the lysosomes; however, its prominence in this organelle increased over time with the majority of the bLF647 signal located in lysosomes. This is in contrast to Tf488 where there was little colocalisation after 1 h with the probe itself recycling out of the cell by the next timepoint (Figure 4B). Some dextran had reached the lysosome after 1 h and there was then an increased and, by 24 h, substantial Dex488-647 colocalisation (Figure 4C).

**Figure 5:**
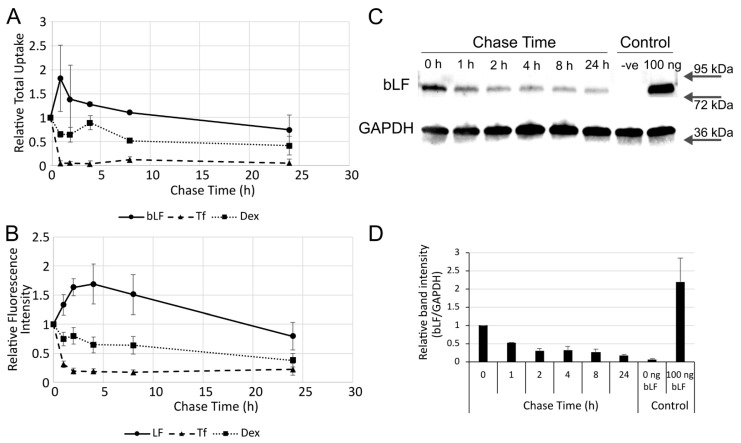
Quantification of trafficking. Cells were incubated with 500 nM bLF647, 100 µg/mL Dex647, or 20 nM Tf488 for 1 h in serum-free medium before being washed and incubated for between 0 and 24 h in serum-containing medium and imaged by confocal microscopy (**A**), flow cytometry (**B**), or Western blotting ((**C**,**D**), 1 µM unlabelled bLF). To obtain relative total uptake (**A**), 10 images were quantified from each independent experiment and normalised to the 0 h chase period. Flow cytometry represents the mean of the median fluorescence from each independent experiment performed in duplicate (N = 3).

To determine the potential rates of exocytosis/recycling of the internalised bLF647, the same experiments used to generate Figure 4 were performed, but with the fluorescence analysed by flow cytometry (Figure 5B). While Tf488 rapidly recycled out of the cell, there was a slower exocytosis of internalised dextran. Similarly, after a 4 h chase time, there was a slow reduction in fluorescence intensity of internalised bLF647; however, there was an unexpected increase between 0 h and this timepoint. A very similar effect can be seen when quantifying cellular fluorescence in the microscopy images (Figure 5A). Further experiments were performed as above with the collection of cell lysates at different chase timepoints. The lysates were separated by SDS-PAGE, and Western blot analysis was performed to detect bLF. The data showed a rapid reduction in detectable bLF signal over the first 2 h of the experiment, with little subsequent reduction until the 24 h timepoint (Figure 5C,D).

## 4. Discussion

In this study, various endocytic aspects of bLF were investigated and, at times, compared with well-characterised markers of endocytosis, with particular interest in understanding bLF cellular dynamics and how these could relate to its antimicrobial activity. For bLF to be considered as a therapeutic, it needs to be nontoxic, and here we show little evidence of loss of viability in A549 cells after 24 h incubation. This supports previous research that showed low toxicity in these cells after 24 h at concentrations <10 µM; however, at high doses (~180 µM) and after 48 h incubation, there was evidence of toxicity [32]. Toxicity has also been studied in breast cancer cell lines where the growth rate was reduced most prominently after more than 24 h incubation at >15 µM bLF; interestingly, little toxicity was seen in the near-normal cell line MCF10-2A [10]. In vivo experiments have, however, shown bLF to be nontoxic in rats up to 2000 mg/kg/day [33].

Both protein receptors and cell surface proteoglycans have been implicated in the binding and uptake of LF. Both an excess of unlabelled bLF and the strong negative charge of heparin were able to significantly reduce, but not eliminate, the cell surface binding of bLF. This is in contrast to transferrin, where excess unlabelled transferrin almost totally reduced Tf488 binding, while heparin had no effect. This suggests that the binding and endocytosis of this protein are more complex, reflecting the fact that the protein can interact with a variety of receptors in different cell lines, whether they be proteins or carbohydrates. While the canonical LfR (also known as intelectin-1) is not expressed in A549 cells, other implicated receptors, such as LDLR, are. Additionally, in line with several studies on CPPs, the hLF N-terminus showed a strong affinity to heparan sulphate, with this being implicated in its endocytosis [34]. This region has also been shown to be important for the internalisation of hLF via the LfR in Caco-2 colon epithelial cells [35], but it remains to be determined whether this alone mediated the uptake of bLF in A549 cells. Transferrin, like LF, is an iron-binding protein which shares ~60% protein sequence similarity with LF [36]; these differences are evidenced in this N-terminal region but also contribute to LF being more cationic the Tf.

Comparative analysis of bLF endocytosis and endocytic trafficking, compared with transferrin and dextran as receptor-mediated and fluid-phase probes, respectively, identified some unique characteristics for the protein we focused on here. Dextran uptake was linear against concentration and time, while transferrin uptake eventually saturates the receptor where cell-associated fluorescence signal plateaus. bLF displayed linear uptake versus concentration, indicating a fluid phase uptake pathway; however, against time, there was an increasing rate of uptake. This could be due to a stimulatory process occurring during uptake or, more likely, it could be a dequenching effect of the fluorophore attached to the protein. In summary, uptake of bLF proved to be more similar to the fluid-phase endocytosis rather than being a receptor-mediated uptake. Interestingly, bLF was shown to internalise through a receptor-mediated process in hepatocytes [3], potentially via LfR, which was not expressed in the A549 cells studied here. Clathrin-mediated endocytosis was shown to be the main internalising factor of both holo- and apo-lactoferrin in Caco-2 cells where the LfR is also expressed [37]. Clathrin was also hypothesised to be the main endocytic pathway in THP-1 macrophage cells [38]; however, there was a depletion in uptake using inhibitors of other endocytic pathways, confirming our studies showing that it can internalise via different receptors and pathways.

Lactoferrin has been researched as an antiviral and antimicrobial agent; however, little research has been performed on where it localises in the cell once it has been internalised, or whether it persists to potentially act against the pathogen. To determine this, we looked at three complimentary techniques to assess the trafficking properties of bLF: confocal microscopy, flow cytometry, and Western blotting. Both flow cytometry and Western blotting showed a persistence of the bLF647 fluorescence signal up to 24 h, indicating they had not been recycled. This does not however refute the possibility that the fluorophore has become decoupled and continues to provide a signal. This is in contrast to Tf488, where the signal was lost following a 1 h chase period, indicating that rapid recycling had occurred. Dex647 showed a gradual loss of signal to ~50% intensity during the 24 h period, indicating that there was a slow expulsion of the fluorophore. An unexpected increase in fluorescence signal was observed in cells incubated with bLF647 after just a 1 h chase by both confocal microscopy and flow cytometry. The reason for this is unclear, but we hypothesise that this may be due to a dequenching effect of the fluorophore as has been seen previously in our studies using Cy5-mRNA-loaded lipid nanoparticles in one cell line [39] and Alexa488-labelled exosomes [40]. However, Western blot analysis of unlabelled bLF did show that there was a decrease in cell-associated bLF over the first 2 h of chasing. This indicated that the majority of the bLF was degraded in lysosomes in 2 h, rather than being exocytosed, and that the remaining fluorescent signal shown by flow cytometry and confocal microscopy emanated from the fluorophore alone or attached to bLF peptides that could not be detected by Western blotting.

In conclusion, our studies show that the protein is effectively internalised, trafficked to lysosomes, and degraded in this lung cell, and provide no evidence that it can reach the nucleus, at least not at the concentrations studied here. It remains to be determined whether the protein, as an intact entity residing at any particular point on its endocytic traffic, can act as an intracellular antimicrobial agent, as we have previously shown for CPPs [41].

## Data Availability

The data presented within this study are available within the article; Cancer Cell Line Encyclopaedia (CCLE) data sets can be found at https://sites.broadinstitute.org/ccle/ (accessed on 27 January 2022).

## Supplementary Materials

The following are available online at https://www.mdpi.com/article/10.3390/pharmaceutics14040855/s1. Table S1: Expression in A549 cells of receptors implicated in LF uptake, Figure S1: Binding of transferrin to A549 cells, Figure S2: Trafficking of bLF647 to lysosomes by confocal microscopy, Figure S3: Trafficking of Tf488 to lysosomes by confocal microscopy, Figure S4: Trafficking of Dex647 to lysosomes by confocal microscopy. References [30,42,43,44,45,46,47] are cited in the supplementary materials.
