# Supplementary material for: Fluid-Phase Endocytosis and Lysosomal Degradation of Bovine Lactoferrin in Lung Cells
Source: Pharmaceutics. 2022 Apr 13;14(4):855. doi: 10.3390/pharmaceutics14040855 (PMC9032238; doi:10.3390/pharmaceutics14040855)
Supplement: Supplementary file 1 [file pharmaceutics-14-00855-s001.zip › pharmaceutics-1624625-supplementary.pdf]

**Fluid phase endocytosis and lysosomal degradation of bovine lactoferrin in lung cells**

**Supplementary Materials**

**Table S1: Expression in A549 cells of receptors implicated in bLF uptake.**

| Receptor | Ligand and/or Competitor | Gene Code | mRNA expression | | | Ref |
|---|---|---|---|---|---|---|
| | | | RNAseq value | % Rank compared to lung Cells | % Rank compared to all cells | |
| LDLR | LDL | LRP1 | 1.78 | 44.5% | 36.9% | [1,2] |
| LRP2/Megalin | Insulin/albumin/ haemoglobin | LRP2 | -7.70 | 33.5% | 37.3% | [2] |
| Intelectin-1 | Arabinogalactan/Lactoferrin | ITLN1 | -13.00 | 0.0% | 0.0% | [3] |
| TLR4 | LPS and others | TLR4 | -7.41 | 15.1% | 11.2% | [4] |
| CXCR4 | SDF1 | CXCR4 | -2.96 | 31.9% | 28.9% | [5] |
| CD14 | LPS | CD14 | -0.63 | 58.1% | 70.9% | [6] |

RNAseq data obtained from CCLE [7] showing receptors and how A549 cells rank in respective receptor expression relative to cells designated as lung or relative to all cell lines in the database.

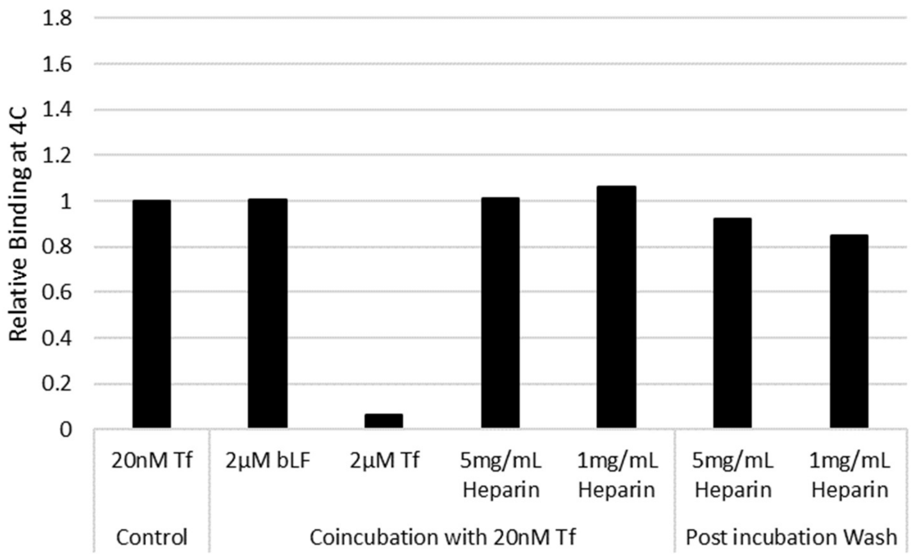


**Figure S1: Binding of transferrin to A549 cells.**
20 nM Tf488 was incubated with A549 cells for 1 h in the presence of 100X molar excess of unlabelled bLF or Tf, Samples were also co-incubated with 5 or 1 mg/mL heparin or washed after the 1 h incubation. Values are calculated relative to Tf488 uptake alone by flow cytometry, N=1 in duplicate.

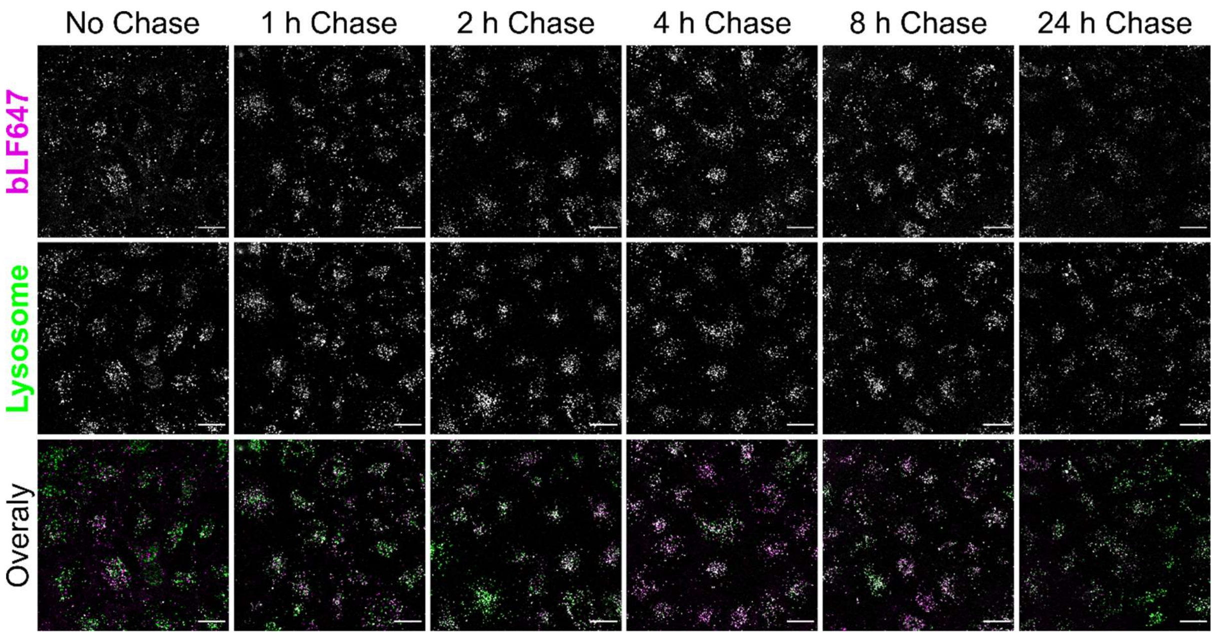


**Figure S2: Trafficking of bLF647 to lysosomes by confocal microscopy.**
Cells were incubated with 500 nM bLF647 (magenta, top row) for 1 h in SFM, washed in serum containing medium and incubated for the chase period before being washed and imaged by confocal microscopy. Lysosomes (green, middle row) were pre-labelled using a pulse-chase protocol. Scale bar = 20 µm, representative images from three independent experiments quantified in Figure 5.

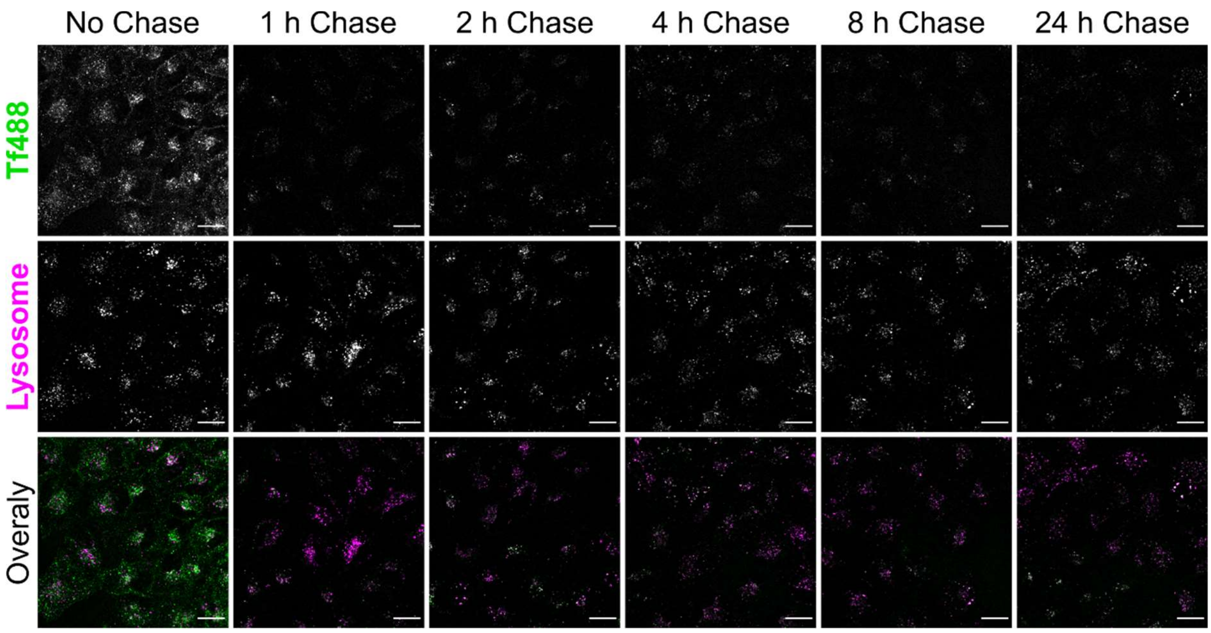


**Figure S3: Trafficking of Tf488 to lysosomes by confocal microscopy.**
Cells were incubated with 20 nM Tf488 (magenta, top row) for 1 h in SFM, washed in serum containing medium and incubated for the chase period before being washed and imaged by confocal microscopy. Lysosomes (green, middle row) were pre-labelled using a pulse-chase protocol. Scale bar = 20 µm, representative images from three independent experiments quantified in Figure 5.

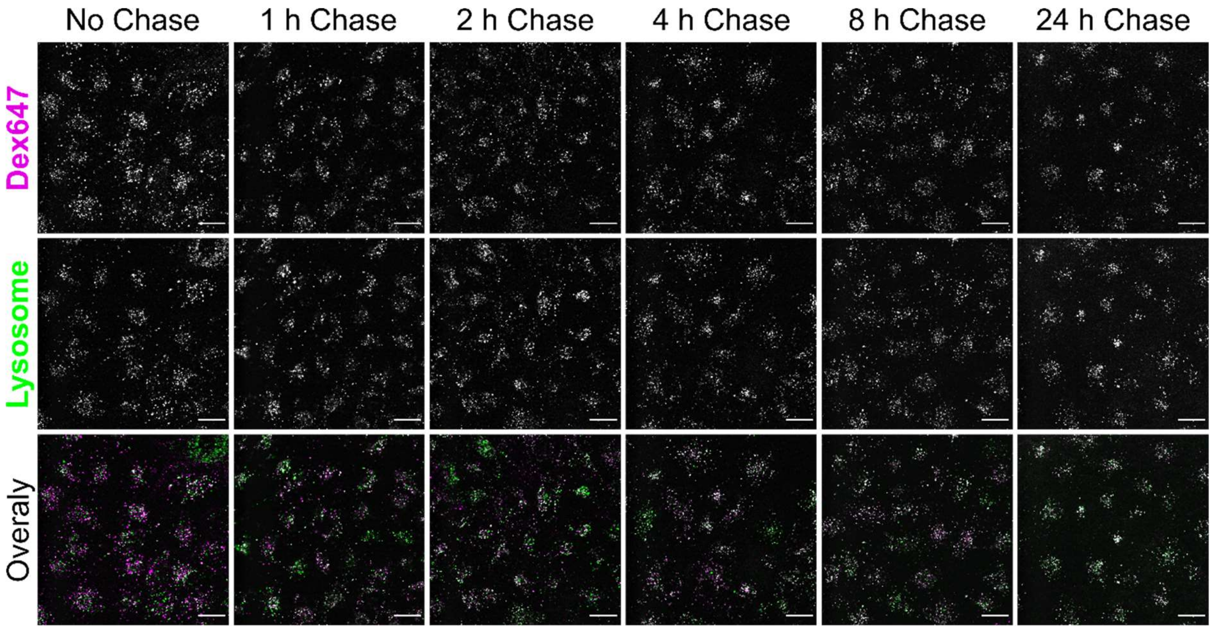


**Figure S4: Trafficking of Dex647 to lysosomes by confocal microscopy.**
Cells were incubated with 50 µg/mL Dex647 (magenta, top row) for 1 h in SFM, washed in serum containing medium and incubated for the chase period before being washed and imaged by confocal microscopy. Lysosomes (green, middle row) were pre-labelled using a pulse-chase protocol. Scale bar = 20 µm, representative images from three independent experiments quantified in Figure 5.

## References


1. Chien, Y.J.; Chen, W.J.; Hsu, W.L.; Chiou, S.S. Bovine lactoferrin inhibits Japanese encephalitis virus by binding to heparan sulfate and receptor for low density lipoprotein. *Virology* **2008**, *379*, 143-151, doi:10.1016/j.virol.2008.06.017.
2. Grey, A.; Banovic, T.; Zhu, Q.; Watson, M.; Callon, K.; Palmano, K.; Ross, J.; Naot, D.; Reid, I.R.; Cornish, J. The low-density lipoprotein receptor-related protein 1 is a mitogenic receptor for lactoferrin in osteoblastic cells. *Mol Endocrinol* **2004**, *18*, 2268-2278, doi:10.1210/me.2003-0456.
3. Shin, K.; Wakabayashi, H.; Yamauchi, K.; Yaeshima, T.; Iwatsuki, K. Recombinant human intelectin binds bovine lactoferrin and its peptides. *Biol Pharm Bull* **2008**, *31*, 1605-1608, doi:10.1248/bpb.31.1605.
4. Curran, C.S.; Demick, K.P.; Mansfield, J.M. Lactoferrin activates macrophages via TLR4-dependent and -independent signaling pathways. *Cell Immunol* **2006**, *242*, 23-30, doi:10.1016/j.cellimm.2006.08.006.
5. Takayama, Y.; Aoki, R.; Uchida, R.; Tajima, A.; Aoki-Yoshida, A. Role of CXC chemokine receptor type 4 as a lactoferrin receptor. *Biochem Cell Biol* **2017**, *95*, 57-63, doi:10.1139/bcb-2016-0039.
6. Perdijk, O.; van Neerven, R.J.J.; van den Brink, E.; Savelkoul, H.F.J.; Brugman, S. Bovine Lactoferrin Modulates Dendritic Cell Differentiation and Function. *Nutrients* **2018**, *10*, doi:10.3390/nu10070848.
7. Nusinow, D.P.; Szpyt, J.; Ghandi, M.; Rose, C.M.; McDonald, E.R., 3rd; Kalocsay, M.; Jane-Valbuena, J.; Gelfand, E.; Schweppe, D.K.; Jedrychowski, M.; et al. Quantitative Proteomics of the Cancer Cell Line Encyclopedia. *Cell* **2020**, *180*, 387-402 e316, doi:10.1016/j.cell.2019.12.023.
